# Influence of Workplace management initiatives on talent engagement in the Nigerian pharmaceutical industry

**DOI:** 10.12688/f1000research.23851.2

**Published:** 2020-11-10

**Authors:** Hezekiah O. Falola, Opeyemi O. Ogueyungbo, Oluwatunmise O. Ojebola

**Affiliations:** 1 Business Management Department, Covenant University, Ota, Ogun State, 112233, Nigeria

**Keywords:** Recognition, wellbeing, learning and development, diversity and inclusion, talent engagement

## Abstract

**Background**: Talent engagement is increasingly gaining the attention of pharmaceutical industry, particularly in developing nations like Nigeria. The existing literature shows that the subject of workplace management initiatives and talent engagement in the Nigerian pharmaceutical industry has not been sufficiently researched. This study investigates the influence of workplace management initiatives on talent engagement in some selected pharmaceutical companies in Nigeria.

**Methods**: In total, 600 respondents were surveyed across various departments and units of ten selected pharmaceutical companies in Nigeria using multiple sampling techniques. Only 429 copies of the questionnaire, representing a 71.5% response rate, were returned and analyzed using Smart PLS 3.0.

**Results:** The outcomes of the statistical analysis show that recognition, employees’ wellbeing, learning and development as well as diversity and inclusion had significant influence on talent, emotional, cognitive and behavioural engagements.

**Conclusions**: In line with the statistical results, the study concludes that workplace management initiatives influenced talent engagement. The study emphasized the need for the review of many workplace management initiatives in order to determine its suitability within the context of pharmaceutical industry in Nigeria.

## Introduction

Most organizations in the 21 ^st^ century experience stiff competitions that necessitates continuous changes currently observed in the global business environment. Organizations, particularly the pharmaceutical companies are now more concerned with fostering sustainable talent engagement initiatives ( Ikon & Chika, 2017). Talent engagement is believed to be sustained through strategic management tactics that motivate and stimulate high and productive talent engagement ( Falola *et al.*, 2018a). In this regard, workplace management initiatives could play a more prominent role in driving talent engagement in the pharmaceutical industry.

It has been established in the literature that management initiatives drive employee engagement and high performance ( Falola *et al.*, 2018a; Ikon & Chika, 2017). Previous studies in this context identified various forms of management initiatives that drive work engagement. For instance, Oludayo *et al.* (2018) studied work-life balance and flexible work arrangements as management initiatives that can be adopted in the banking sector. Falola *et al.* (2020a) examined various forms of initiatives provided by the management of institutions of higher learning in driving job effectiveness such as research grants, conference support initiative, and pedagogy support among others. Falola *et al.* (2018a) also studied the influence of work engagement initiatives as predictors of academic staff behavioral outcomes in Nigeria Universities. Other researchers also studied diversity management initiatives, workplace stress management initiatives, emotional intelligence initiatives, and organizational energy initiatives among others ( Aguwa *et al.*, 2014; Ibidunni *et al.*, 2019; Onyokoko *et al.*, 2019; Oseni, 2017). Most of these studies were carried out mostly in institutions of higher learning, the banking industry, the fast moving consumer goods (FMCGs) manufacturing sector and the public health sector in Nigeria. This suggests that there is knowledge gap in the area of workplace management initiatives and talent engagement in Nigeria pharmaceutical industry. This current study investigates the influence of workplace management initiatives (recognition, wellbeing, learning and development as well as diversity and inclusion) on talent engagement (talent emotional engagement, talent cognitive engagement and talent behavioural engagement) in the pharmaceutical industry in Nigeria.

Moreover, most existing empirical studies used correlation and regression for the analysis of the collected and coded data, however, the present study adopts Smart PLS 3.0 as the statistical tool for the analysis. This gap in the literature is given attention in this current study by looking at the influence of workplace management initiatives on talent engagement in the pharmaceutical industry in Nigeria with the following specific objectives. The specific objectives are to:

i. investigate the influence of employee outstanding performance recognition on talent engagement;
ii. examine the effect of employees’ wellbeing on talent engagement;
iii. determine the effect of learning and development initiatives on talent engagement and
iv. investigate the influence of diversity and inclusion on talent engagement.

The insight offers by this study can potentially help the management of the pharmaceutical industry in Nigeria to improve on the workplace management initiatives that will enhance talent engagement in the industry. The findings of this study can be leveraged by management and other stakeholders in the industry for policy formulation and implementation that will enhance talent engagement.

## Literature review

### Employee recognition and talent engagement

Recognition is the informal, timely or formal acknowledgement of an individual or team's effort. If efforts of employees are adequately recognised with appropriate performance incentives, employees’ engagement will be enhanced ( Falola *et al.*, 2018b). Nazir & Islam (2017) classified employee recognition into four major types, these include personal recognition, result recognition, work practice recognition and job dedication recognition. Generally, everyone wants to be recognised. Masri & Elsuliman (2019) argued that the most important form of employee recognition is work practice recognition. Findings reveal that lack of work practice recognition is very risky because it contributes to workplace distress ( Masri & Elsuliman, 2019; Nazir & Islam, 2017).

Recognizing employee’s achievement sends a positive signal of being valued and cared for by the organizational management which engenders their dedication and engagement to organizational goals ( Kahn, 1990). This is reflected in their acquisition of more knowledge and experiences (talent cognitive engagement), display of positive feelings and emotions towards their jobs, supervisors and colleagues (talent emotional engagement) and in physical actions put into jobs (talent behavioural engagement). Inegbenojie (2018) reiterated that employee recognition is a management tool used to achieve talent engagement in organizations. Workplace management initiatives such as recognition and employee wellbeing foster employees’ engagement in the world of work.

### Employee wellbeing and talent engagement

Wellbeing according to the World Health Organization (2020) is defined as the state of good physical health, mental, emotional, social and financial wellness. Wellbeing is the state of employee’s state of happiness, self-actualization, life satisfaction and positive mood in the workplace. Studies have shown that if employees are healthy, satisfied and emotionally stable, they will be more productively engaged ( Falola *et al.*, 2018b; Bakker *et al.*, 2014; Smith & Smith, 2019). Thus, for organizations to earn employee’s engagement, their wellbeing must be prioritized. This can be done by putting in place initiatives in the areas of their financial, health, educational and financial wellness ( Keeman *et al.*, 2017). The financial wellness initiatives assist employees to manage their daily finances and achieve long-term financial goals. Organizations do offer financial counseling in the areas of budgeting, managing assets, building credit, reducing debt and saving for retirement to ensure employee financial wellness ( Keeman *et al.*, 2017).

Some organizations provide employees with access to earned wages based on demand to help them solve cash flow problems, especially for low-wage workers ( Bennett *et al.*, 2017). All these are some of the initiatives that motivate employees to be more engaged in the world of work. Educational wellness on the other hand is a management initiative to encourage employees to further their education through full or partial scholarships ( Bennett *et al.*, 2017). Organizations also help to improve employee’s health status by conducting regular health screenings and tests to deal with preventable and chronic conditions ( World Health Organization, 2020). Studies have shown that employees with high level of health, financial and educational wellbeing put in positive thought and effort into their job responsibilities in the work place ( Keeman *et al.*, 2017; Saks, 2016).

### Learning/development and talent engagement

Learning and development is organizational consistent effort in enhancing employee acquisition of knowledge, job-related skills and improved employee behaviour. Employees are able to acquire, transfer, interpret and share information better and more engaged with their core job responsibilities ( Chiekezie *et al.*, 2016). Learning and development is an essential administrative function of human resource management (HRM) that helps the employees to be productively engaged. According to Adelere (2017) employees are the most important assets of any organization, superseding money, materials and machines. According to Siddiqui (2019) employee learning and development is considered as the most essential function of any proficient organization, including those in the pharmaceutical industry. As a result, the pharmaceutical industry has invested heavily in employee learning and development to operate the machines used for drug production processes, which in turn better facilitates employee engagement which is a major driver of competitive advantage ( Ogueyungbo *et al.*, 2019).

### Diversity/inclusion and talent engagement

Diversity in the organization is not tantamount to chaos and reduced output as a creative and engaged workforce with diverse ideas and talents can foster the accomplishment of organizational goals ( Onwuchekwa *et al.*, 2019). Diversity brings people of different ages, skills, language, education, values, competences, beliefs, sexual orientation, race, disability, religion, tribe, culture, gender, colour, ethnicity, nationality, lifestyle and economic status together ( Akinnusi *et al.*, 2017).

Management initiatives of managing diversity/inclusion is an organizational strategy used to foster an encouraging workplace environment. Diversity management initiatives must recognise, respect, tolerate and accept differences among employees ( Akinnusi *et al.*, 2017). Talent engagement according to Macey *et al.* (2019) is determined by many interactions in the workplace, one of which is diversity. Scholars like Onwuchekwa *et al.* (2019); Macey *et al.* (2019) asserted that employee engagement is dependent upon various factors in the workplace. Nevertheless, Inegbenojie (2018) opine that individual differences and effective inclusive management approach play a vital role in determining an employee's level of engagement.

## Methods

### Research design

The study used a survey for data collection which was performed between November 2018 and January 2020.

The study adopted descriptive research design. The descriptive survey design approach focuses on the phenomenon of interest which is intended to provide reliable answers to questions about the measurement of variables. The choice of survey method was based on its time effectiveness as well as its general picture of respondents’ opinions which enhances hypotheses testing. This helped the authors to investigate whether workplace management initiatives had any relationship with talent engagement in some selected pharmaceutical companies in Nigeria. This study adopted the positivism research philosophy. What informs the choice of positivism is based on the application of knowledge is based on natural phenomena, their properties and relationships. Positivism thus prefers quantitative approach rather than qualitative model in data collection with a view to establishing trends and patterns. To this end, the study used quantitative research approach.

### Sample size and sampling procedures

There are 115 registered pharmaceutical companies in Nigeria out of which over 50% are located in Lagos State ( Pharmapproach, 2018). The ten best rated pharmaceutical companies were selected in Lagos State Nigeria. Lagos State was chosen because of the high concentration of pharmaceutical companies in the state. In addition, all the ten selected companies manufacture their products locally. The selection of the ten pharmaceutical companies was based the year of establishment, the number of brands, growth and expansion, the total number of products manufactured, and the corporate image ( Pharmapproach, 2018; PMG-Man, 2019). Also, it is believed that the best rated pharmaceutical companies must have been managing and retaining the talented employees over the years. The retention and engagement of the employees with distinctive talents must have culminated into the level of performance, scientific innovation and quality service delivery of the selected companies. The justification for the criteria used are presented as follows:

i. The selected companies are credible indigenous pharmaceutical organizations that have distinguished themselves in the pharmaceutical industry and have been in existence for a minimum of 40 years with uninterrupted production.
ii. They produce a minimum of 100 branded pharmaceutical products to their names, ranging from tablets, caplets, oral liquid syrups and other consumables.
iii. They also produce a minimum of 1 billion tablets, 250 million capsules, 20 million powder sachets annually.
iv. They have a presence in all the geo-political zones in Nigeria where they either produce, market or distribute pharmaceutical products that meet international standards to government at all levels and private health care providers.
v. The criteria for determination of the corporate image include: (1) recognizable brand, (2) familiarization of people with the companies, (3) the level of partnership, and (4) perceived opinion of customers and employees of the selected companies.

The ten selected pharmaceutical companies have a total 3787 employees excluding casual, contract and lower level staff which represents the population of the study. Sample size was determined using Bartlett *et al.* (2001) table chart and the sample size accounted for 570 and was approximated to 600 to allow for unreturned copies of administered questionnaire at the margin of error of 0.05. Proportional affixation criterion (PAC) was used for the determination of the copies of questionnaire administered to each organization. Pharmaceutical companies sample in each stratum is proportional to the relative weight of the study population as depicted in Table 1.

**Table 1.** Breakdown of selected pharmaceutical companies.

Pharmaceutical companies	Population of the categorised Employees	Sample size	Return rate of administered questionnaire
**Pharm A**	371	57	39
**Pharm B**	380	60	47
**Pharm C**	314	50	34
**Pharm D**	317	50	35
**Pharm E**	421	67	46
**Pharm F**	442	70	52
**Pharm G**	368	58	40
**Pharm H**	343	54	39
**Pharm I**	390	78	53
**Pharm J**	341	54	44
**TOTAL**	**3,787**	**600**	**429**

Multiple samplings (purposive, stratified and simple random) techniques were used in this study. Purposive sampling was used because only the permanent employees at the middle, senior and executive management level cadres with a minimum of 2 years in their current organizations participated in the survey in order to get more accurate and reliable information. Also, stratified sampling was adopted because the population comprises different strata and all employees in each stratum were giving equal chance of been selected with the use of a simple random sampling method. The study used a structured questionnaire adapted from the existing literature to collect data from the respondents with the use of 5-point Likert scale. Copies of questionnaire were administered by the research staff with the help of three research assistants during working hours.

### Measures and variables

To measure workplace management initiatives, the authors created a workplace management initiatives (WMI) scale by adapting the elements of Cannon (2015); Inegbenojie (2018) and Keeman *et al.* (2017) instruments. The researchers developed the measurement instruments into 12 items representing the four domains of (1) recognition, (2) employee wellbeing, (3) learning and development, and (4) diversity and inclusion. Each of the domain was measured with three items each on a 5 Likert scale of strongly agree (5), agree (4), indifference (3), disagree (2), and strongly disagree (1). Based on the response options categorization, the higher total scores on the scale indicate respondents' perception on the subject of workplace management initiatives The following are the examples of the items: “My organization takes time to publicly acknowledge my successes”, “My organization provides incentives, bonuses and other rewards for outstanding performance”, “I receive praise from my supervisor when I successfully reach performance goals or other targets”, “My organization gives adequate attention to employees’ wellbeing”, “The welfare packages for employees are motivating”, “My organization gives priority to safety of lives and property of the employees”, “My organization provides conducive environment that encourages learning and development”, “I have access to the learning and development I need to do my job well”, “This is great company for me to make a contribution to my development”, “Employees who are different from most others are treated fairly in my organization”, “My organization is committed to diversity and inclusion”, “In my organization, everyone has access to equal employment opportunities regardless of their differences”.

To measure talent engagement, the authors adapted the elements of Nazir & Islam (2017) instruments in developing the items. The measurement instruments were developed into nine items representing the three domains of (1) cognitive engagement, (2) behavioural engagement, and (3) emotional engagement. Each of the domain was measured with three items each on a 5 Likert scale of strongly agree (5), agree (4), indifference (3), disagree (2), and strongly disagree (1). Based on the response options categorization, the higher total scores on the scale indicate respondents’ perception on the subject of talent engagement. The examples the items are: “My job allows me to be creative in performing my responsibilities”, “My organization encourages me to use my skills and capabilities in the work process”, “I often think of ways and suggestions to improve my work”, “I feel a sense of belonging and identify with my organization”, I am proud to work in this organization”, “I feel like staying in this organization”, “I exert a lot of energy doing work”, “I feel energized and empowered at work”, “I stay at work till the job is finished”. Face and content validity of the items were checked while internal consistency reliability testing was carried out through composite reliability, average variance extracted (AVE) estimate as well as Cronbach's Alpha. A copy of the questionnaire is provided as extended data ( Falola *et al.*, 2020b).

### Ethical consideration

The principal investigator submitted an application for ethical approval of research proposals to the Business Management Research Ethics Committee on 9 ^th^ October 2018 for approval (Approval No: BMREC/18/21/102). Research team was given introduction letter that was presented to the selected organizations stating the purpose of the research. All the participants were adequately informed about the objective of this study. Only willing employees participated in the survey. All respondents were assured they would stay anonymous and assured that their responses will be treated with extreme confidentiality. It is equally imperative to note that verbal consent was gotten from the respondents of this research. The representatives of the selected pharmaceutical companies were consulted for research permission guidelines. Based on the information provided in principle, an application letter was written requesting permission to research their organizations with the objective of the study clearly stated. Also, the research ethics approval form was attached to the application letter. This type of research is categorised as exempt research that involves a survey with no or minimal risk i.e. level 1 research as presented in the Research Ethical Application Form. In social and management sciences, exempt research does not require signed consent from the participants rather implied consent is usually sufficient particularly in the spirit of anonymity and confidentiality. Through verbal consent, the researchers ensured that the respondents were well informed about the context and intent of this research and were kept abreast with the process and regime of participation.

### Validity and reliability of the research instrument

The research instrument validity was determined through face and content validity by two distinguished professors in the field. Meanwhile factor loadings, compose reliability, average variance extracted (AVE) estimate as well as Cronbach Alpha were carried out to ascertain the reliability of the research instrument. A pilot study was conducted to ascertain the validity and reliability of the research instrument. As recommended by Baker (1994) the sample size for pilot study should be at least 10% of the study population. The sample size of the main study was expected to be 600 using Bartlett *et al.* (2001) table chart. In total, 60 copies of questionnaire were administered to a pharmaceutical company in Ota. The pilot result shows that data was normally distributed and the scale reliabilities (factor loadings, compose reliability, average variance extracted (AVE) estimate as well as Cronbach’s Alpha) were well above the benchmarks as recommended by Byrne (2001) and Tabachnick & Fidell (2007). The statistical outcomes are depicted in Table 2.

**Table 2.** Confirmatory Factor Analysis.

		Loading	AVE	Compose Reliability	Cronbach’s Alpha	RhO.A
Constructs		**> 0.7**	**>0.5**	**> 0.8**	**> 0.7**	
Recognition (R)			0.615	0.864	0.791	0817
	R1	0.765				
	R2	0.890				
	R3	0.754				
Employees Wellbeing (EW)			0.642	0.877	0.813	0.824
	EW1	0.774				
	EW2	0.800				
	EW3	0.870				
Learning and Development (L&D			0.564	0.837	0.739	0.756
	LD1	0.845				
	LD2	0.757				
	LD3	0.732				
Diversity & Inclusion (DI)			0.700	0.903	0.857	0.858
	DI1	0.846				
	DI2	0.832				
	DI3	0.837				
Talent Engagement (TE)			0.526	0.816	0.701	0.702
	TE1	0.722				
	TE2	0.710				
	TE3	0.743				

### Statistical analysis

Statistical Package for Social Sciences (SPSS) software version 22 was used for the coding of the retrieved data while Smart PLS 3.0 was used for the analysis of the data that shows the degree of influence of workplace management initiatives on talent engagement in the pharmaceutical industry. The smart PLS displays the algorithm and bootstrapping models. Algorithm model is a structure of regressions in terms of weight vectors that helps to determine the path co-efficient, the r-square values and the significance values. The bootstrapping helps in determining the significance testing of coefficient and t-values. The default bootstrapping in Smart PLS is 500 subsamples which helps to facilitate significant results. This was enhanced by increasing bootstrapping value to 5000 as suggested by ( Ramayah *et al.* (2018). Meanwhile, linear regression analysis can as well be used as alternative statistical tool for the analysis. Also, the processes and procedures required for the assumptions of credible research analysis were systematically and carefully checked to be sure that the data presented are sufficiently adequate and accurate as recommended by Kline (2005). The assumptions include, appropriate research design, reliabilities, data normality and linearity. In total, 429 copies of questionnaire representing (71.5%) response rate were returned and used as final sample for this study.

## Results

**Respondents profile**: Gender: Male 249 (58%), Female 180 (42%); Age: Most of the respondents (284; 66%) were between 31–50 years; Marital Status: Single 111 (25.9%), Married 286 (66.7%) others 32 (7.5%); Work experience: Most of the respondents (330; 77%) had between 6-20 years work experience; Level: Middle management 236 (55%), Senior management 169 (38.9%), Executive management 26 (6.1%).

**Figure 1.**
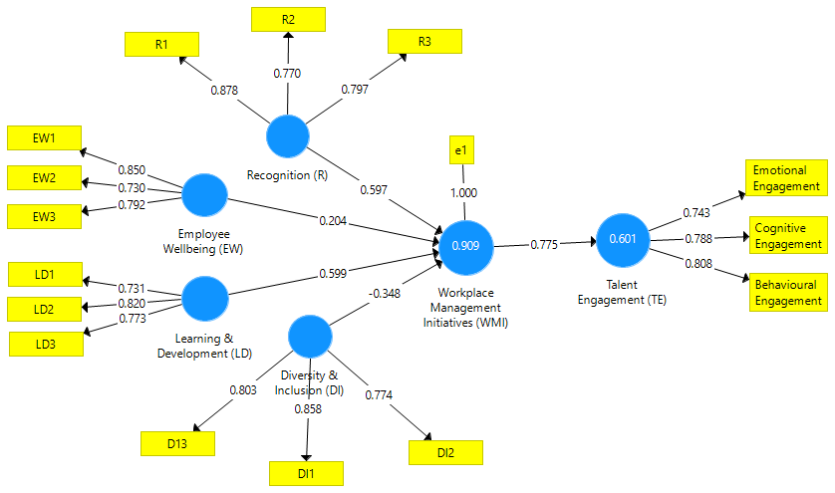
PLS algorithm model of workplace management initiatives and talent engagement. *PLS: partial least squares, PLS Algorithm is used for formative scales. R: recognition, EW: employee wellbeing, LD: learning & development, DI: diversity & inclusion*.

Workplace management initiatives were measured with four constructs: recognition, wellbeing, learning and development, and diversity and inclusion while affective engagement, behavioural engagement, cognitive were used to measure talent engagement. For the purpose of the interpretation of the analysis, R-Square i.e the coefficient of determination, structural path co-efficient (B value), T-statistic value, and P-values are critical indicators of Smart PLS used for the determination of the results as depicted in Figure 1– Figure 3 ( Falola *et al.*, 2020b).

**Figure 2.**
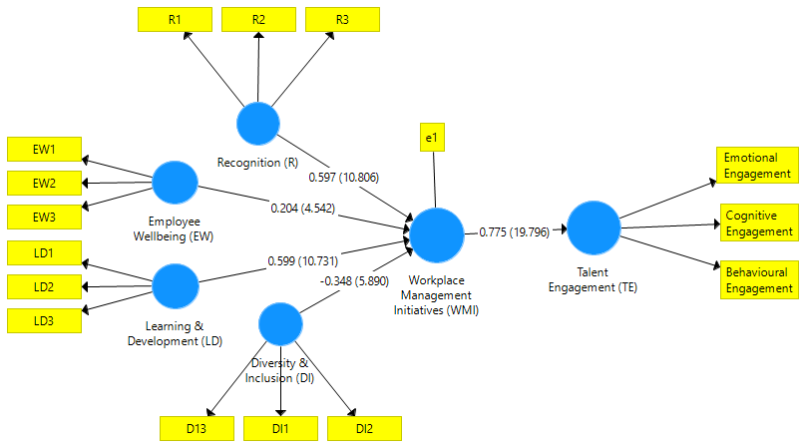
PLS Bootstrapping Model with β and T values of workplace management initiatives and talent engagement. *PLS: partial least squares, β: beta value, T values: calculated differences represented in units of standard error, R: recognition, EW: employee wellbeing, LD: learning & development, DI: diversity & inclusion*.

**Figure 3.**
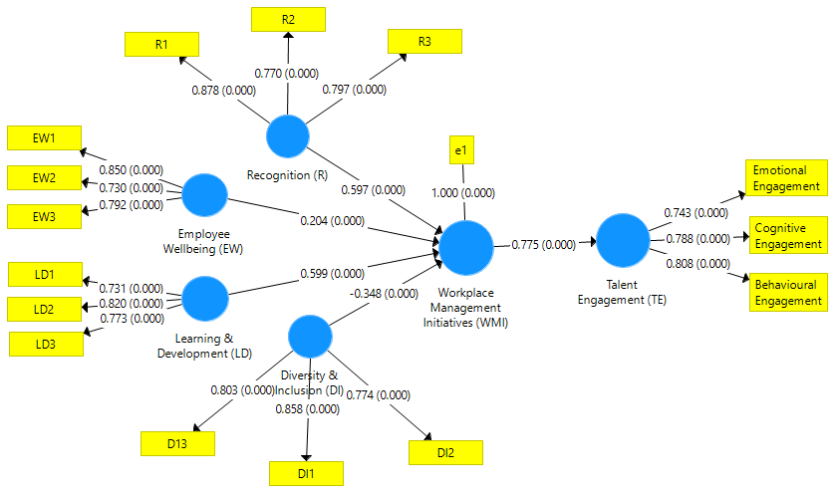
PLS Bootstrapping Model with β and P values of workplace management initiatives and talent engagement. *PLS: partial least squares, β: beta value, P values: calculated probability, R: recognition, EW: employee wellbeing, LD: learning & development, DI: diversity & inclusion*.

Figure 1 depicts PLS algorithm model of workplace management initiatives that drives talent engagement. The path depicts the degree of relationship between the observed variables. The R-square which determines the level of variance of talent engagement and workplace management initiatives. R-square could be substantial (>0.75), moderate (>0.50) or weak (>0.25.). Figure 1 shows that the R ^2^ = 0.909 for workplace management initiatives. This implies that all the factor loading items of talent engagement weakly but significantly explain 90.9% of the variance in workplace management initiatives. In a related development, Figure 1 also indicates that talent engagement collectively explained 60.1% of the variability of talent engagement. This implies that the items of workplace management initiatives explain 60.1% of the variance in talent engagement.

Bootstrapping is recommended by Ramayah *et al.* (2018) was used to evaluate T-values. We also reran the analysis with the bootstrapping setting to 5000 as suggested by Henseler *et al.* (2012) and Garson (2016) for confirmatory purposes. Bootstrapping helps in calculating path coefficients, outer loading, outer weights, indirect effect and total effect as depicted in Figure 2. All the T-values presented in Figure 2 are above 1.96 while the p-values presented in Figure 3 are significant at 0.05. This suggests that workplace management initiatives have significant influence on talent engagement.

The β value which indicates the expected variance in talent engagement for a unit variation in the workplace management initiatives was used to test the significance of the formulated hypotheses. The greater the β value the more the substantial effect on workplace management initiatives. The significant effect of workplace management initiatives on talent engagement was verified through the T-statistical test. The path co-efficient of the observed variables are presented in Table 3 ( Falola *et al.*, 2020b).

**Table 3.** Path co-efficient of workplace management initiatives and talent engagement.

Variables	Path Co- efficient	Indirect Effect	Standard Deviation	T Statistics	P Values
**Workplace Mgt. Initiatives (WMI) → Talent** **Engagement (TE)**	**0.775**	**0.687**	**0.039**	**19.796**	**0.000**
R 1 → WMI	0.597		0.054	10.806	0.000
R2 → TE		0.158	0.036	4.427	0.000
EW1 → WMI	0.204		0.044	4.542	0.000
EW2 → TE		0.158	0.036	4.427	0.000
LD 1 → WMI	0.599		0.057	10.731	0.000
LD 2 → TE		0.464	0.046	9.978	0.000
DI1 → WMI	0.348		0.060	5.890	0.000
DI2 → TE		0.279	0.044	6.135	0.000
R Square					
		R Square		Adjusted R Square	
**Workplace Mgt. Initiatives (WMI)**		0.909		0.905	
**Talent Engagement (TE)**		0.601		0.596	

## Discussion

Further to the PLS statistical and empirical results presented in Table 3, it was observed that the structural path co-efficient of the measures of workplace management initiatives i.e recognition, employee wellbeing, learning and development and diversity and inclusion indicate significant relationship at 0.05. Similarly, the path co-efficient also revealed that employee outstanding performance recognition initiatives (R1) indirectly and significantly influenced talent engagement (β=0.463, T-value=4.427, P-value =0.000 <0.05). This implies that, if an employee outstanding performance recognition initiative is given priority by the management of pharmaceutical industry it will significantly enrich talent engagement. This finding corroborates Masri & El-suliman (2019) submission that recognition of employee outstanding performance through various performance incentives plays a significant role in the level of talent engagement. This suggests that for the pharmaceutical industry in Nigeria to enhance talent engagement, recognition of optimal and excellent performance should be given priority. This also supports the findings of ( Inegbenojie, 2018) who noted that employee recognition of performance as a result of training provided enhances job engagement.

The path co-efficient value shows that employee wellbeing has an indirect significant influence on talent engagement (β=0.158, T-value=4.427, P-value =0.000 <0.05). This implies that employee wellbeing, if strengthened by the management of the pharmaceutical companies, will enhance talent engagement. This finding validates the submission of Keeman *et al.* (2017) and the World Health Organization (2020). The finding also corroborates the findings of Bennett *et al.* (2017) that if management are concerned about the wellbeing of their workforce, it will naturally motivate employees to be emotionally and cognitively attached to the organization with a sense of job satisfaction. Saks (2016) noted that if employees are satisfied with their job, the level of their engagement will be enhanced.

Similarly, learning and development also have indirect significant influence on talent engagement (β=0.464, T-value=9.978, P-value =0.000 <0.05). This finding validates the submissions of ( Adelere, 2017; Chiekezie *et al.*, 2016; Siddiqui, 2019). They found out that learning and development will increase the level of talent engagement considerably. The implication of this is that if management of the pharmaceutical companies invest in the learning and development of employees, their level of exposure to the best practices in their various specialisation will be enhanced. As noted by Siddiqui (2019) well exposed employees are likely to be more productively engaged.

In a related development, it was also revealed from the path co-efficient that diversity and inclusion has an indirect significant influence on talent engagement (β=0.270, T-value=6.135, P-value =0.000 <0.05). This implies that diversity and inclusion initiatives of the management in the pharmaceutical industry will promote and enhance talent engagement. This corroborates with the findings of Onyokoko *et al.* (2019) and Emeh *et al.* (2017). Akinnusi *et al.* (2017) also validates this finding and posited that diversity and inclusion give employees a sense of belonging. As noted by Schneider *et al.* (2017), if employees perceived that they have a sense of belonging, they tend to be more engaged. Table 3 also shows that learning and development has the highest beta value among other measures of workplace management initiatives that best predict talent engagement. This suggests that management of the pharmaceutical companies should continue to invest in the learning and development initiative that drives talent engagement. Largely, the path co-efficient shows that the level of influence of workplace management initiatives on talent engagement is statistically significant with a beta value of 0.775 with T-Statistical value of 19.815. This implies that workplace management initiatives have significant influence on talent engagement. The beta value of 0.775 suggests 77.5% influence on principal variable i.e. if one unit of workplace management initiatives increases, then 77.5% talent engagement will increase.

### The practical implication of the study

The study investigated the influence of workplace management initiatives on talent engagement in Nigerian pharmaceutical industry. Employees from reputable pharmaceutical firms formed the respondents’’ base for the study. This study posits that dimensions of workplace management initiatives, especially recognition of exceptional job performance, employee wellbeing, learning and development as well as diversity and inclusion are significant to influence the level of engagement of employees with distinctive competencies in the pharmaceutical industry. It is recommended that management of the pharmaceutical industry should invest in organisational supports through appropriate and timely welfare initiatives that will foster talent engagement. Moreover, management must understand their roles as professional leaders and influencers towards the employees in driving optimal talent engagement. The role of the leadership of the pharmaceutical industry, especially with regards to ensuring workplace initiatives that will continuously create an enabling and conducive work environment that permit employees to stay abreast of best practices and get engaged with their job responsibilities is also strongly advised. All the stakeholders in the pharmaceutical industry are admonished to support initiatives that will encourage productive engagement of the employees.

## Conclusion

Workplace management initiatives in driving talent engagement in Nigeria pharmaceutical industry cannot be overemphasised. Therefore, investing in workplace initiatives that drive talent engagement can be regarded as a strategic approach to promoting engagement. Recognition of excellent performance through performance incentives, employee wellbeing packages, relevant learning and development initiatives as well as diversity and inclusion are some of the workplace management initiatives that can be leveraged by the management of the pharmaceutical industry in Nigeria to enhance talent emotional, cognitive and behavioural engagement.

## Limitations and suggestions for further studies

This study covers only ten outstanding pharmaceutical companies in Lagos, Nigeria. This implies that the scope of the selected organization is not wide-ranged. Therefore, future studies may broaden the scope of the study to include other pharmaceutical companies in other major cities and zones in Nigeria. This study investigated the influence of workplace management initiatives on talent engagement. Only four constructs such as recognition, wellbeing, learning and development as well as diversity and inclusion were used for the measurement of workplace management initiatives, future study can add to the number of constructs an also introduce moderating variable that may help in strengthening relationships between workplace management initiatives and talent engagement. Future study can also look at the possibility of using mixed method as against the quantitative method used in this current study for a more elaborate finding.

## Data availability

### Underlying data

Figshare: SPSS DATA-WMI.sav. https://doi.org/10.6084/m9.figshare.12589571.v1 ( Falola *et al.*, 2020b)

This project contains the following underlying data:

- SPSS DATA-WMI & TE.sav (SPSS data on workplace management initiatives and talent engagement)

### Extended data

Figshare: SPSS DATA-WMI.sav. https://doi.org/10.6084/m9.figshare.12589571.v1 ( Falola *et al.*, 2020b)

This project contains the following extended data:

- QUESTIONNAIRE-WMI & TE.docx (Study questionnaire)

Data are available under the terms of the Creative Commons Zero “No rights reserved” data waiver (CC0 1.0 Public domain dedication).
