# Supplementary material for: Influence of Workplace management initiatives on talent engagement in the Nigerian pharmaceutical industry
Source: F1000Res. 2020 Nov 10;9:699. Originally published 2020 Jul 13. [Version 2] doi: 10.12688/f1000research.23851.2 (PMC7676391; doi:10.12688/f1000research.23851.2)
Supplement: Peer review history

# Peer review: Reviewer response for version 2

**DOI:** 10.5256/f1000research.30535.r74518

**Published:** 2020-12-08

**Authors:** Deepika Pandita

**Affiliations:** 1 Symbiosis Institute of Business Management, Symbiosis International University, Pune, India

All the reviews are incorporated.

Is the work clearly and accurately presented and does it cite the current literature?

Partly

If applicable, is the statistical analysis and its interpretation appropriate?

Yes

Are all the source data underlying the results available to ensure full reproducibility?

Partly

Is the study design appropriate and is the work technically sound?

Partly

Are the conclusions drawn adequately supported by the results?

Partly

Are sufficient details of methods and analysis provided to allow replication by others?

Yes

Reviewer Expertise:

human resources, talent management, employee engagement

I confirm that I have read this submission and believe that I have an appropriate level of expertise to confirm that it is of an acceptable scientific standard.

# Peer review: Reviewer response for version 2

**DOI:** 10.5256/f1000research.30535.r74517

**Published:** 2020-11-17

**Authors:** Yejide Olukemi Oseni

**Affiliations:** 1 Pharmacists Council of Nigeria, Ibadan, Nigeria; 2 Obafemi Awolowo University, Ile-Ife, Nigeria

The author had revised all the areas pointed out. However, I will still like to know why the use of "affective engagement" in RESULTS para 2, line 2?

Is the work clearly and accurately presented and does it cite the current literature?

Yes

If applicable, is the statistical analysis and its interpretation appropriate?

Yes

Are all the source data underlying the results available to ensure full reproducibility?

Yes

Is the study design appropriate and is the work technically sound?

Partly

Are the conclusions drawn adequately supported by the results?

Yes

Are sufficient details of methods and analysis provided to allow replication by others?

Yes

Reviewer Expertise:

Pharmacy administration, public health, health and pharmacy policy and regulation, pharmacy practice, health management

I confirm that I have read this submission and believe that I have an appropriate level of expertise to confirm that it is of an acceptable scientific standard.

# Peer review: Reviewer response for version 2

**DOI:** 10.5256/f1000research.30535.r74516

**Published:** 2020-11-17

**Authors:** Cecile Schultz

**Affiliations:** 1 People Management Department, Tshwane University of Technology, Pretoria, Gauteng, South Africa

The article can now be accepted and published.

Is the work clearly and accurately presented and does it cite the current literature?

Yes

If applicable, is the statistical analysis and its interpretation appropriate?

Yes

Are all the source data underlying the results available to ensure full reproducibility?

Yes

Is the study design appropriate and is the work technically sound?

Yes

Are the conclusions drawn adequately supported by the results?

Yes

Are sufficient details of methods and analysis provided to allow replication by others?

Yes

Reviewer Expertise:

Organisational behaviour and human resource management

I confirm that I have read this submission and believe that I have an appropriate level of expertise to confirm that it is of an acceptable scientific standard.

# Peer review: Reviewer response for version 1

**DOI:** 10.5256/f1000research.26318.r73152

**Published:** 2020-10-28

**Authors:** Deepika Pandita

**Affiliations:** 1 Symbiosis Institute of Business Management, Symbiosis International University, Pune, India

- **Introduction**: 1. Though the main objective is stated explicitly specific objectives need to be highlighted. 2. Objectives need to be written carefully with suitable words like examine, investigate will make your research more important.  
- **Literature review**: The literature review needs to be arranged well. Is your definition of job engagement the same as work engagement and employee engagement? The entire sequencing of the constructs in the literature review needs to be re-examined.  
- **Methods**: Create subheadings and emphasize study design, study area, study period, sample size, research instrument, data collection method, and analysis of the data.
  You have not mentioned the study design in your paper. The profile of the respondent is also not stated. Sampling frame and sample size should be a part of the research design. Why were the best rated pharma companies chosen? Any rationale behind that?
- ** Results**: Results needs to be presented carefully. 
  There is no mention of practical implications in your study. 

Is the work clearly and accurately presented and does it cite the current literature?

Partly

If applicable, is the statistical analysis and its interpretation appropriate?

Yes

Are all the source data underlying the results available to ensure full reproducibility?

Partly

Is the study design appropriate and is the work technically sound?

Partly

Are the conclusions drawn adequately supported by the results?

Partly

Are sufficient details of methods and analysis provided to allow replication by others?

Yes

Reviewer Expertise:

human resources, talent management, employee engagement

I confirm that I have read this submission and believe that I have an appropriate level of expertise to confirm that it is of an acceptable scientific standard, however I have significant reservations, as outlined above.

# Response

**Published:** 2020-10-29

**Authors:** Hezekiah Falola

**Affiliations:** Covenant University, Nigeria

We want to sincerely appreciate Deepika Pandita for the insightful comments and suggestions. 

 All the issues raised have been effected in version 2 of the article.

 Thank you

# Peer review: Reviewer response for version 1

**DOI:** 10.5256/f1000research.26318.r73158

**Published:** 2020-10-28

**Authors:** Cecile Schultz

**Affiliations:** 1 People Management Department, Tshwane University of Technology, Pretoria, Gauteng, South Africa

- An interesting and technically neat study was presented. Recent and relevant sources were used to discuss the variables of the study.
- The gap in the literature study was clear.
- Appropriate statistics were used to investigate the research objective.
- Discussions were elaborative and supported by previous studies.
- The authors must elaborate on the research philosophy(positivism perhaps?), research design (survey research design) and the research approach (quantitative).
- The practical implications of the study must also be addressed.

Is the work clearly and accurately presented and does it cite the current literature?

Yes

If applicable, is the statistical analysis and its interpretation appropriate?

Yes

Are all the source data underlying the results available to ensure full reproducibility?

Yes

Is the study design appropriate and is the work technically sound?

Yes

Are the conclusions drawn adequately supported by the results?

Yes

Are sufficient details of methods and analysis provided to allow replication by others?

Yes

Reviewer Expertise:

Organisational behaviour and human resource management

I confirm that I have read this submission and believe that I have an appropriate level of expertise to confirm that it is of an acceptable scientific standard, however I have significant reservations, as outlined above.

# Response

**Published:** 2020-10-29

**Authors:** Hezekiah Falola

**Affiliations:** Covenant University, Nigeria

Cecile Schultz is deeply appreciated for reviewing our article. The methodology has been revised as suggested and the implications of the study has been included in version 2 of the article  as recommended.  

 Thank you

# Peer review: Reviewer response for version 1

**DOI:** 10.5256/f1000research.26318.r72011

**Published:** 2020-10-12

**Authors:** Yejide Olukemi Oseni

**Affiliations:** 1 Pharmacists Council of Nigeria, Ibadan, Nigeria; 2 Obafemi Awolowo University, Ile-Ife, Nigeria

Title could read: "Influence of workplace management initiatives on talent engagement in Nigerian Pharmaceutical Industry."

**Introduction:**

1. The specific objectives if need be stated should follow the main objective earlier stated.
2. I could not see the reason for the specific objectives.
3. The use of different adjectives to capture the specific objectives has no meaning in the work done i.e. Investigate the influence of …, examine the effect…., determine the effect…
4. Is using different analytical tools a gap? Are you saying the tool has never been used for a similar study in the past?

**Literature review:**

1. For flow in your discussion and alignment with your stated objectives, rearrange the discussion sub-head accordingly.
2. Is job engagement the same as work engagement and employee engagement?
3. Under **“Talent** wellbeing and job engagement” subhead, corrected to “ **Employee **wellbeing and job engagement”.
4. “Employee recognition and talent engagement” subhead could be better captioned as “Employee performance and talent engagement”.
5. The last statement under “ **Talent** wellbeing and job engagement” and “Employee performance and talent engagement” subheads look like your conclusion. I think should be expunged.
6. The second paragraph under “Diversity/ inclusion and talent management” subhead is a mix-up. 1 ^st^ sentence looks like another conclusion. 3 ^rd^ sentence could have been stated at the beginning of the subhead if necessary. 4 ^th^ sentence is not necessary at all because that was what you were discussing.

**Methods:**

1. You need to streamline your methods to show every important aspect of it: study design, study area, study period, sample size, research instrument, data collection method, data analysis, and ethical approval.
2. Your study design is not stated.
3. **Respondent Profile** as stated should be presented in the **Result**. Hence the Section A of the Questionnaire which is sociodemographic characteristics of the respondents should be discussed in the research instrument.
4. The last two statements in the **sample size and sampling procedure** should be part of your research instrument. Also the Pharmacists Council of Nigeria Register is the only official document that will give you an accurate number of manufacturing premises registered for that year.
5. Why did you use the ten best-rated pharmaceutical companies only?
6. Bullet iii under justification for criteria have two points merged.
7. **Inclusion and exclusion criteria** is a tautology. You have discussed it under **sample size and sampling procedure.**
8. **Measures and variables **are Section B of your research instrument. Present it with Section A.
9. The way this statement “The organisation encourages me to use my skills and capabilities in the work process” is drafted makes it more of a workplace management initiative measure rather than talent engagement measurement. Also this statement “This is great company for me to make a contribution to my development” is more of a talent engagement measure.

**Results:**

1. Result of the sociodemographic characteristics as presented in the **Respondent Profile **should come here.
2. Line 3; “affective” used for the first time here. Be consistent. I think you mean emotional.

** Limitations:**

1. Separate limitations and suggestions for further studies

Is the work clearly and accurately presented and does it cite the current literature?

Yes

If applicable, is the statistical analysis and its interpretation appropriate?

Yes

Are all the source data underlying the results available to ensure full reproducibility?

Yes

Is the study design appropriate and is the work technically sound?

Partly

Are the conclusions drawn adequately supported by the results?

Yes

Are sufficient details of methods and analysis provided to allow replication by others?

Yes

Reviewer Expertise:

Pharmacy administration, public health, health and pharmacy policy and regulation, pharmacy practice, health management

I confirm that I have read this submission and believe that I have an appropriate level of expertise to confirm that it is of an acceptable scientific standard, however I have significant reservations, as outlined above.

# Response

**Published:** 2020-10-15

**Authors:** Hezekiah Falola

**Affiliations:** Covenant University, Nigeria

Yejide Olukemi Oseni is deeply appreciated for taking her time to review our article. Her comments and observations are valued. Our responses to some of the issues raised are presented below:

**Introduction**

The authors decided to operationalize the independent and dependents variables to allow the readers to know the coverage of the constructs used for the measurement of both the independent and dependent variables. Therefore, the specific objectives that stemmed from the broad objective were carefully presented in the study.

**Literature Review**

Job engagement, work engagement, employee engagement, and talent engagement are similar concepts that can be used interchangeably, particularly in HRM parlance.

Also, talent in HRM means employees with distinctive competencies. Therefore, talent wellbeing is more appropriate within the context of the study.

In HRM employee recognition is not the same as employee performance. The influence of employee compensation in the prediction of talent engagement was the focus here. Therefore, the authors would like to retain the original subhead, i.e. Employee recognition and talent engagement.

**Methodology**

The robust methodology is one of the areas that F1000Research emphasized for article publication consideration. Streamlining the methodology aspect of the study might not be necessary. Besides, the authors explained all the essential elements of the research methods using appropriate justifications. Also, since the sociodemographic characteristics of the respondents were not part of the objective of the study, the authors decided to present the summary of the respondents' profile which was believed to be sufficient for the study. 

**Results**

The dimensions of engagement as established in the literature are affective, behavioural and cognitive, among others. Using affective is not out of place, particularly in this type of study. 

**Limitations**

Since this is an article and not a thesis or dissertation, separating limitations and suggestions for future studies might not be necessary.
